# Systemic Treatment with Pioglitazone Reverses Vision Loss in Preclinical Glaucoma Models

**DOI:** 10.3390/biom12020281

**Published:** 2022-02-09

**Authors:** Huilan Zeng, Alina V. Dumitrescu, David Wadkins, Benjamin W. Elwood, Oliver W. Gramlich, Markus H. Kuehn

**Affiliations:** 1 Department of Ophthalmology, Second Xiangya Hospital, Central South University, Changsha 410011, China; huilan-zeng@uiowa.edu; 2 Department of Ophthalmology and Visual Sciences, The University of Iowa, Iowa City, IA 52242, USA; alina-dumitrescu@uiowa.edu (A.V.D.); david-wadkins@uiowa.edu (D.W.); benjamin-elwood@uiowa.edu (B.W.E.); oliver-gramlich@uiowa.edu (O.W.G.); 3 Iowa City VA Center for the Prevention and Treatment of Visual Loss, Iowa City, IA 52246, USA; 4 Human Clinical Research Center of Ophthalmic Disease, Changsha 410011, China

**Keywords:** glaucoma, PPARγ, pioglitazone, glucose metabolism

## Abstract

Neuroinflammation significantly contributes to the pathophysiology of several neurodegenerative diseases. This is also the case in glaucoma and may be a reason why many patients suffer from progressive vision loss despite maximal reduction in intraocular pressure. Pioglitazone is an agonist of the peroxisome proliferator-activated receptor gamma (PPARγ) whose pleiotrophic activities include modulation of cellular energy metabolism and reduction in inflammation. In this study we employed the DBA2/J mouse model of glaucoma with chronically elevated intraocular pressure to investigate whether oral low-dose pioglitazone treatment preserves retinal ganglion cell (RGC) survival. We then used an inducible glaucoma model in C57BL/6J mice to determine visual function, pattern electroretinographs, and tracking of optokinetic reflex. Our findings demonstrate that pioglitazone treatment does significantly protect RGCs and prevents axonal degeneration in the glaucomatous retina. Furthermore, treatment preserves and partially reverses vision loss in spite of continuously elevated intraocular pressure. These data suggest that pioglitazone may provide treatment benefits for those glaucoma patients experiencing continued vision loss.

## 1. Introduction

Glaucoma is the leading cause of irreversible blindness in the world. The disease is characterized by optic nerve head changes, narrowing of the visual field and decreased vision caused by dysfunction and death of retinal ganglion cells (RGCs) [1,2]. Reducing the intraocular pressure (IOP) in patients by medical or surgical means is currently the only approved treatment and while this approach does reduce the rate of disease progression, vision continues to be lost in many patients [3]. These individuals would benefit from neuroprotective regimens that directly target RGCs, but despite considerable research efforts such therapies are not currently available. The difficulty in developing effective neuroprotective treatments in glaucoma is due, at least in part, to the multifactorial etiology of the disease. Factors including disturbances in ocular blood flow, immune-mediated processes, oxidative stress, metabolic vulnerability, and genetic predisposition have all been implicated in causing the disease [4,5,6,7,8,9].

Loss of RGCs is the ultimate cause of vision loss and to date these cells cannot be replaced. However, prior to apoptotic cell death, RGCs undergo distinct morphological changes, including loss of dendritic complexity and reduction in soma size [10]. These changes are, at least in part, reversible and a reduction in IOP has been shown to increase contrast sensitivity in patients and animal models of glaucoma [11,12,13].

RGC distress and demise in the glaucomatous retina initiate chronic retinal neuroinflammation that plays an important role in the subsequent pathology of the disease [7,14,15,16]. Chronic neuroinflammation as a pathogenic mechanism has been observed in several other progressive neurodegenerative diseases, including Alzheimer’s disease, Parkinson’s disease, cerebral ischemia, and traumatic brain injury [17,18,19]. Symptoms of these conditions appear to be ameliorated by treatment with pioglitazone, a thiazolidinedione-type insulin sensitizing drug that has been extensively used to treat Type II diabetes [20,21]. While the main indication for pioglitazone use is glycemic control, the drug readily crosses the blood–brain barrier and therefore exerts its activities on the central nervous system and the retina [22,23].

The exact mechanism through which pioglitazone affords protection in neurodegenerative diseases has not been conclusively determined, but it is well known that this drug acts as an agonist for peroxisome proliferator-activated receptor gamma (PPARγ), a member of the nuclear hormone receptor family. The activation of PPARγ can exert powerful anti-inflammatory effects through its inhibition of the NF-κB and mTOR pathways [24,25]. Data from multiple laboratories have demonstrated that similar pathomechanisms are also involved in glaucoma [26,27], suggesting that pioglitazone therapy may benefit glaucoma patients and provide neuroprotection for RGCs. In this study we evaluate the ability of pioglitazone to morphologically protect RGCs and optic nerve axons and to preserve visual function in two distinct pre-clinical mouse models of glaucoma.

## 2. Materials and Methods

### 2.1. Animals

All animal experiments have been approved by the University of Iowa and the Iowa City VA IACUC and were conducted in accordance with the ARVO Statement for the Use of Animals in Ophthalmic and Vision Research (protocol numbers 6101877 and 1790601). C57BL/6J (B6) and DBA/2J mouse breeder pairs were obtained from The Jackson Laboratory (Bar Harbor, ME, USA) and housed in the animal facility of the Iowa City VAMC on a 12 h light–dark cycle (6 a.m. to 6 p.m.) with food ad libitum. Equal numbers of both sexes were used in our experiments, and all animals were euthanized by CO_2_ inhalation followed by cervical dislocation.

### 2.2. Pioglitazone Treatment

Pioglitazone was given to the group of drug-treated mice in the drinking water, whereas the control group received water without pioglitazone. Tablets were ground into powder and dissolved in water to prepare a 15 mg/100 mL aqueous solution. The solution was replaced every three days. DBA2/J and C57BL/6J consume approximately 6 mL of water/day [28] and consequently each mouse received approximately 0.9 mg/day or 3.6 mg/100 g/day.

### 2.3. Induction of Elevated Intraocular Pressure (IOP)

Elevated IOP was induced in C57BL/6J mice by injections of the adenoviral vector Ad5RSVmyocillinY437H (Ad5myoc, University of Iowa Viral Vector Core, Iowa City, IA, USA) as previously described [29,30,31]. Briefly, newborn (P2–P4) B6 mice received a subcutaneous injection of Ad5myoc (3 × 103 PfU) to induce tolerance to the vector and prevent ocular inflammation. At eight weeks of age, 4.8 × 108 pfU virus in 3 µL PBS was delivered into the anterior chamber of the eye by transcorneal injection. The development of IOP was monitored using a rebound tonometer (Tonolab, Colonial Medical Supply, Windham, NH, USA) in isoflurane-sedated mice as described previously [32]. The IOP measurements were performed between 9 a.m. and 1 p.m. by a researcher who was blind to the animals’ treatment condition. Animals that did not have an increase in intraocular pressure above the baseline by at least 7 mm Hg for at least 12 weeks were excluded from the analysis.

### 2.4. Immunohistochemical Quantitation of Retinal Ganglion Cells

Mice were sacrificed, immediately enucleated, and the eyes were fixed in 4% paraformaldehyde for one hour. The eyes were infiltrated with increasing concentrations of sucrose and embedded in Optimal Cutting Temperature (OCT) compound (Fisher Scientific, Waltham, MA, USA). Seven-micron thick sagittal sections were prepared on a cryostat and blocked with 5% BSA/0.03% Triton-X100 in PBS for one hour at room temperature. Retinas were incubated with rabbit anti-gamma synuclein antibodies (1:100 dilution, Abcam ab55424, Cambridge, MA, USA) at 4 °C overnight on a rocker platform [33]. After washes in PBS, binding was visualized following incubation in a donkey anti-rabbit Alexa Fluor 488 secondary antibody solution for three hours at room temperature in the dark. Retinas were extensively washed in PBS and then mounted with Vectashield (Vector Laboratories, Burlingame, CA, USA). All sections were counterstained with the nuclear dye DAPI (Invitrogen, Carlsbad, CA, USA) to facilitate orientation and viewed on an Olympus (Melville, NY, USA) BX41 microscope equipped with a SPOT RT camera (Diagnostic Instruments; Sterling Heights, MI, USA). Gamma synuclein positive RGCs were counted in twelve non-consecutive sections per eye.

### 2.5. Evaluation of Optic Nerve Damage

Optic nerve damage was evaluated as described previously [34,35]. Immediately after death, the optic nerve was carefully dissected and a 3 mm long part of the optic nerve, located about 2 mm behind the eyeball, was carefully removed and fixed in 2% glutaraldehyde and 2% paraformaldehyde. After osmium fixation, the tissue was stained with uranyl acetate and embedded in Eponate 12 resin. One-micron thick sections were taken on a plane perpendicular to the length of the optic nerve. The sections were stained with 1% p-phenylenediamine (PPD) and micrographs were taken at 400× magnification. A grading scheme similar to that used by other researchers was used to assess the degree of optic nerve damage and the optic nerve was assigned one of four grades of damage [36,37] as follow. Level 1: Healthy nerves—few (<50) deeply stained (damaged) axons are detected in the entire optic nerve; Level 2: Slight damage—damaged axons are sparse, but to detect (each 5–10% of nerves); Grade 3: Moderate damage—11–40% of axons are damaged, and there may be obvious glial hypertrophy; Grade 4: Severe damage—more than 40% of axons are damaged. The loss of axons is obvious, and swollen axons and/or glial cell proliferation can be detected. The degree of damage was independently assessed by two investigators in a masked manner.

### 2.6. Optokinetic Reflex Measurements

Visual ability in B6 mice was measured using an OptoDrum (StriaTech, Tübingen, Germany). Mice were placed on a platform inside the apparatus and a slowly rotating stripe pattern was generated on computer screens and presented to the animal. Reflexive head movement was monitored by a camera and correct tracking responses to the presented pattern were detected by the system’s software. The spatial frequency of the stimulus is increased or decreased using a staircase method to determine the threshold of visual acuity as cycles per degree (c/d) at 99.8% contrast. Lack of a tracking response indicates that the pattern is no longer perceived and defines the visual ability of the mouse. Trials in each session are repeated until the spatial frequency and direction can be determined unequivocally. In all cases the operator was blinded to the treatment status of the tested animal.

### 2.7. Pattern Electroretinography (pERG) Recording

Mice were anesthetized by intraperitoneal injection of ketamine (30 mg/kg, Mylan, Canonsburg, PA, USA), xylazine (5 mg/kg, Akorn Inc., Lake Forest, IL, USA) and acepromazine (2.3 mg/kg, Rattlesnake Drugs, Scottsdale, AZ, USA). After using 1% tropicamide to dilate the pupil, a drop of GenTeal gel (Alcon Laboratories, Fort Worth, TX, USA) was placed on the corneal surface to maintain corneal integrity. PERG was recorded using a pattern stimulator (Diagnosys Celeris System, Diagnosys LLC, Lowell, MA, USA) at 1 Hz (2 inversions per second) and 50 cd/m² alternating, reverse, black and white vertical stimulation response. Six hundred traces were recorded from each eye, the average waveform was calculated, and the amplitude (µV) from the peak of P1 to the valley of N2 was measured. All recordings were carried out while maintaining the animals’ body temperature between 37 °C and 38 °C using the system’s thermal pad.

### 2.8. Statistical Treatment of Data

All IOP and RGC data are given as mean ± standard deviation (SD) and were tested for Gaussian distribution before statistical analysis. To test for pairwise significance between two groups, the Student’s t-test was used and for statistics including more than two groups, *p* values were calculated using ANOVA followed by Tukey’s post hoc tests. The optic nerve damage grade was compared using the Wilcoxon signed-rank test. All calculations were performed using GraphPad Prism Version 8.1 (GraphPad Software: San Diego, CA, USA) and *p* values < 0.05 were considered statistically significant.

## 3. Results

### 3.1. Pioglitazone Reduces RGC Damage in DBA/2J Mice

The DBA/2J mouse spontaneously develops increased intraocular pressure and damage to RGCs and is among the best characterized experimental glaucoma models [38,39,40]. In this strain, eye abnormalities, including iris atrophy and pupil deformation develop early followed by rising IOP, beginning at 6–7 months of age in most, but not all, eyes. IOP reaches a peak at 9–10 months of age, and then steadily declines [41]. This process is accompanied by the loss of RGCs and the degeneration of optic nerve axons.

**Figure 1:**
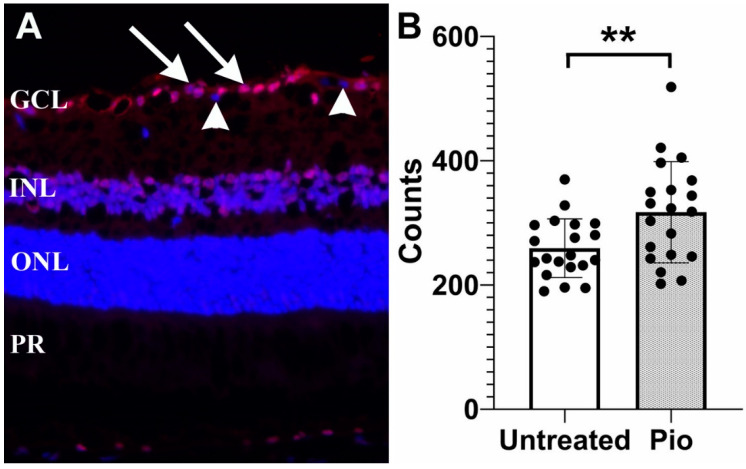
Protective effect of pioglitazone in DBA/2J mice. (**A**) Representative image showing γ-synuclein labeling of RGCs (arrows). Displaced amacrine cells are not bound by this antibody and are not counted (arrowheads) (**B**) RGC density in pioglitazone treated mice (Pio) is significantly higher than in untreated controls (*p* = 0.009). Each dot represents one eye. GCL: Ganglion cell layer; INL: Inner nuclear layer, ONL: Outer nuclear layer, PR: Photoreceptor inner segments. **: *p* < 0.01.

In order to determine if pioglitazone provides a protective effect on RGCs, mice (*n* = 20) were placed on a low-dose regimen of pioglitazone (3.6 mg/100 g/day) beginning at the age of 6 months. A second group of control mice (*n* = 20) were left untreated. All mice were sacrificed at 11 months of age and loss of RGCs was determined (Figure 1). Our findings demonstrated that untreated DBA2/J mice displayed 259.3 ± 47.2 RGC/section, whereas mice treated with pioglitazone displayed a significantly higher number of cells (317 ± 81.4 RGC/section, *p* = 0.009).

**Figure 2:**
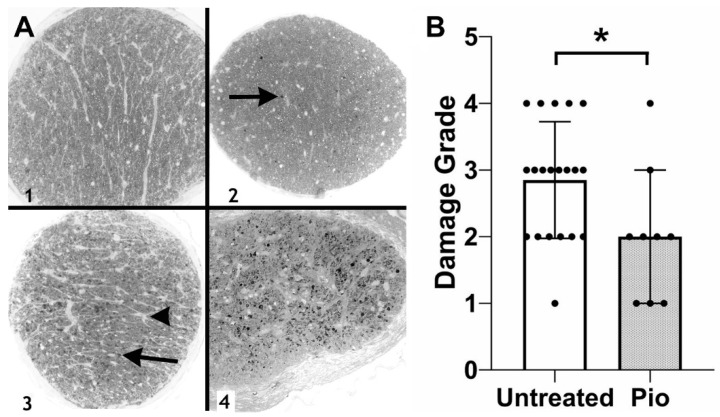
Optic nerve damage in DBA2/J mice. (**A**) Examples of optic nerve damage grades. A grade of 1 indicates a normal optic nerve, whereas a grade of 4 is assigned to optic nerves displaying severe damage and numerous PPD stained axons (arrows) and areas of gliosis (arrowhead). (**B**) Grading of optic nerve damage demonstrates a protective effect in pioglitazone (Pio)-treated mice (*p* = 0.0334). Each dot represents one optic nerve. *: *p* < 0.05.

The finding of reduced RGC damage was confirmed through the analysis of optic nerve damage (Figure 2). Optic nerves obtained from control (untreated) DBA/2J mice (*n* = 20) displayed an average damage grade of 2.85, significantly worse than the pioglitazone-treated group (average: 2.00, *p* = 0.0334, *n* = 9). Together, these data indicate that low-dose pioglitazone treatment prevents RGC degeneration in this glaucoma model.

### 3.2. Ad5myoc Induced Increased IOP in C57/B6 Mice

One of the drawbacks of the DBA/2J model for vision research is the fact that in this strain, pattern electroretinograms (pERG) may be affected by iris adhesion and poor pupil dilation [42]. Furthermore, this strain lacks the optomotor reflex, which is useful to functionally assess vision in rodents [43]. Finally, the determination of IOP is difficult in this strain, possibly due to the development of corneal calcification with age, which affects the accuracy of rebound tonometry. In order to confirm the protective effect and functionally evaluate this finding, we employed a distinct mouse model of glaucoma that is reliant upon adenoviral-mediated expression of a pathogenic variant of human myocilin in the trabecular meshwork of mice [29,30,44].

**Figure 3:**
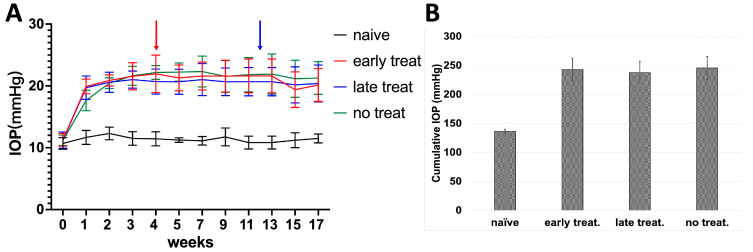
IOP in C57BL/6J mice following injection of Ad5myoc into the anterior chamber. (**A**) IOP profile of treatment groups. Pioglitazone treatment was initiated either 4 weeks (red arrow) or 12 weeks (blue arrow) after induction of elevated IOP. (**B**) Average cumulative IOP in treatment groups. No significant differences in cumulative IOP were observed between Ad5myoc-injected mice (*p* > 0.25).

Here, we injected 9 × 10^7^ pfU Ad5myoc into the anterior chamber of both eyes in three groups of C57BL/6J mice (*n* = 10 mice/group). In addition, an untreated naïve control group was included. Of those mice that received Ad5myoc injections, one group did not receive pioglitazone treatment (no treatment control), while the two other groups received pioglitazone in the drinking water either starting 4 weeks after Ad5myoc injection (early treatment group), or 12 weeks after Ad5myoc injection (late treatment group). During the two weeks following Ad5myoc injection, IOP increased by approximately 9–12 mmHg in injected eyes and remained stable until the end of the experimental period (Figure 3A). A change in IOP upon initiation of pioglitazone treatment was not observed. The cumulative IOP exposure over the experimental period was significantly higher in all Ad5myoc-treated groups than in naïve mice (ranging from *p* = 1.03 × 10^−8^ to *p* = 5.9 × 10^−10^, Figure 3B), but statistically significant differences between those groups were not observed (ranging from *p* = 0.25 to *p* = 0.70). A change in IOP in the naïve control group was not noted.

### 3.3. Pioglitazone Improves Visual Function in a Mouse Model of Glaucoma

**Figure 4:**
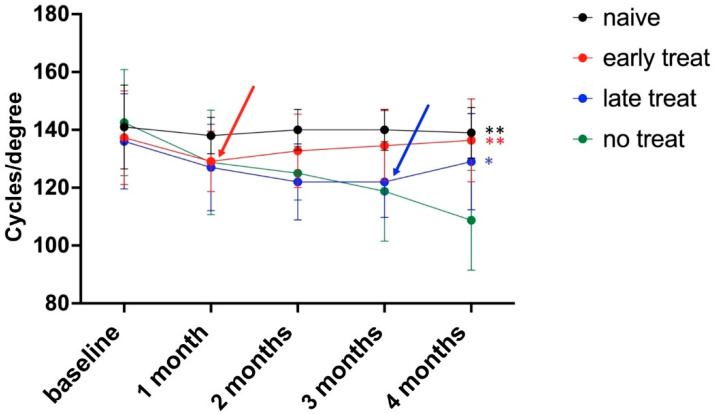
Pioglitazone improves visual acuity in glaucomatous mice. Induction of elevated IOP following baseline measurements leads to a decrease in OKR. Pioglitazone treatment after one month (early treatment, red line) caused a reversal of these deficits. Pioglitazone treatment in mice with more advanced damage (late treatment group, blue line) prevents further functional decline and partially reverses losses. Arrows indicate the start of pioglitazone treatments. ** *p* < 0.01; * *p* > 0.05.

We then determined the visual function in the groups of mice by measuring the optokinetic reflex (OKR). All animals were tested prior to IOP elevation (baseline) to confirm that no undetected visual deficits existed. Overall, contrast sensitivity in these 2-month-old C57BL/6J mice was 138.9 ± 15.9 cycles/degree (c/d) and this value remained unchanged in naïve mice over the next 4 months (139.0 ± 8.8 c/d). Upon elevation of IOP, the OKR noticeably declined, which was already apparent after 1 month in all groups (Figure 4). In untreated mice with elevated IOP the OKR continued to degrade and reached 108.8 ± 17.3 c/d after 4 months of elevated IOP. Animals in the early treatment group received pioglitazone in the drinking water after one month of elevated IOP, which led to a gradual improvement in visual function. At the end of the experimental period these mice displayed a cycle threshold of 136 ± 14.4 c/d, similar to that of naïve mice and significantly better than untreated animals (*p* = 0.0014).

In the late treatment group, glaucomatous damage was allowed to progress for 3 months before pioglitazone treatment was initiated. Thus, these mice received pioglitazone for only 28 days prior to termination of the experiment. Before-treatment visual function in this group deteriorated at a rate similar to that observed in untreated controls with elevated IOP. However, pioglitazone treatment after 3 months of elevated IOP improved the cycle threshold from 122 ± 12.3 c/d to 129 ± 16.6 c/d within 4 weeks and visual function was clearly better than in untreated mice with elevated IOP (*p* = 0.023). When compared to naïve mice without elevated IOP, animals in the late treatment group had somewhat lower visual acuity at the end of the trial, but the differences were not statistically significantly (*p* = 0.11). A difference between male and female animals was not noted [45]. Consequently, pioglitazone treatment not only prevented a further decline in visual function in these mice with existing glaucomatous damage but, as observed in the early treatment group, also caused a slight improvement.

### 3.4. Pioglitazone Preserves Pattern ERG Amplitudes in Mice with Elevated IOP

**Figure 5:**
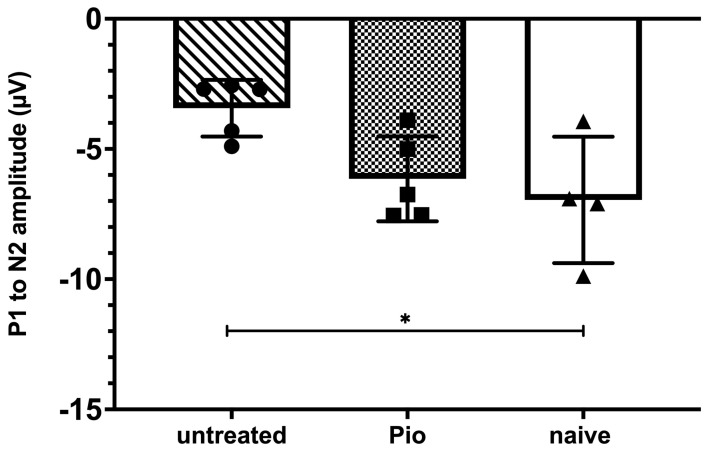
Pattern electroretinography (pERG) after 8 weeks of elevated IOP. The P1 to N2 amplitude is significantly diminished in untreated mice when compared to naïve animals (*p* = 0.028). Animals receiving pioglitazone in the drinking water for 28 days before pERG measurements did not display significant differences when compared to naïve mice (*p* = 0.72). *: *p* < 0.05.

The effect of pioglitazone on RGC function was further evaluated using pERG in randomly chosen subgroups of mice from the early treatment, no treatment, and naïve cohorts. As described above, elevated IOP was induced by Ad5myoc injection and animals in the early treatment group began to receive pioglitazone in the drinking water after one month. pERG measurements were carried out 28 days thereafter, i.e., mice had been exposed to elevated IOP for two months. As expected, the P1 to N2 amplitude of untreated mice with elevated IOP declined significantly when compared to naïve animals (−3.43 ± 1.09 µV vs. −6.96 ± 2.42 µV; *p* = 0.028, Figure 5). In contrast, pioglitazone-treated animals with elevated IOP did not display significant differences to naïve mice (−6.14 ± 1.63 µV; *p* = 0.72) and tended to perform better than untreated glaucomatous mice, although our statistical threshold was narrowly missed (*p* = 0.07).

## 4. Discussion

Pioglitazone has been shown to be beneficial in several neurodegenerative conditions, including Alzheimer’s disease and chronic traumatic brain injury [46,47]. Our study was designed to evaluate whether a protective effect can also be observed in glaucoma. Toward this goal, we used pioglitazone in two genetically and mechanistically distinct pre-clinical mouse models of the disease. In our initial experiment, we administered oral pioglitazone to DBA/2J mice starting at 6 months of age when RGC damage is minimal in this model. Histopathologic evaluation of the eyes 5 months later demonstrated significant preservation of optic nerve integrity in concert with significantly higher RGC survival in pioglitazone-treated animals. The neuroprotective effect was then functionally confirmed in C57BL/6J mice with induced elevation of IOP. Pioglitazone treatment not only prevented further loss of vision but also restored visual acuity. This effect was particularly evident in animals with mild damage (early treatment group) but could also be observed in mice with more advanced glaucoma. The preservation of functional vision is congruent with the notion that pioglitazone treatment results in RGC survival, although this was not directly confirmed histochemically in Ad5.myoc C57BL/6J mice.

While our findings strongly indicate that pioglitazone is not only neuroprotective in glaucoma and also has the potential to reverse some vision loss, the mechanism(s) through which this occurs remain to be elucidated. Pioglitazone exerts pleiotropic effects including acceleration of glucose flux via glycolysis, reduction in circulating levels of inflammatory cytokines, and improving vascular dysfunction [48]. One of the best described effects of pioglitazone is its activity as an agonist of peroxisome proliferator-activated receptor γ (PPARγ), a nuclear receptor involved in the regulation of insulin sensitivity and glucose metabolism [49]. Upon activation, PPARγ binds to specific PPAR response elements (PPRE) and influences the transcription of key genes involved in lipid and glucide metabolism. This may be an important aspect of the observed neuroprotective effects since recent studies have suggested that metabolic disruption may be a cause of glaucomatous neurodegeneration [9,14]. As such, it is conceivable that pioglitazone treatment alters the cellular metabolism of retinal neurons or glia to diminish stress on RGCs. Furthermore, PPARγ agonists also inhibit the activity of the mechanistic target of rapamycin (mTOR) [50]. The inhibition of mTOR then reduces the activity of the mTOR complex 1 (mTORC1), which serves to sense the energy state of cells and regulates protein synthesis, autophagy, mitochondrial function, lipogenesis, ketogenesis, and glucose homeostasis [51]. Inhibition of the complex would further alter the energy state of the cell and foster autophagy which may provide protection to RGCs by heightened removal of aggregated proteins or impaired organelles [52]. It is noteworthy that rapamycin treatment, which also inhibits mTOR, has been shown to be neuroprotective in rats [53] and mice [27].

Alternatively, it is possible that pioglitazone mediated protection of RGCs in glaucoma is the result of decreased retinal neuroinflammation. An increasing body of evidence has demonstrated that innate immune responses are not simply a result of elevated IOP, but can actively contribute to RGC dysfunction and loss in glaucoma [7,54]. Activation of PPARγ inhibits the synthesis of pro-inflammatory genes by reducing the transcriptional activity of NFκB, a central regulator of innate immunity [55]. In the glaucomatous retina, NFκB, is synthesized by astroglial cells and further exacerbates the progression of the disease [26,56].

Finally, our data suggest that pioglitazone treatment increases retinal function despite continued IOP elevation. It is unlikely that pioglitazone causes RGC proliferation and it is more plausible that treatment improves the function of RGCs that are distressed, but surviving. Elevated IOP causes a number of morphologic and molecular adaptations to RGCs that may aid their survival, but reduce their functional state. One example is the disruption of GABAergic signaling that occurs rapidly after IOP elevation and causes a decrease in the pERG signal [30,57]. It is conceivable that resolution of either metabolic or neuroinflammatory stress through PPARγ activity reverses the dysregulation and leads to the observed increase in retinal function. Additionally, retinal function may be increased through a pioglitazone-induced increase in insulin signaling, which may play a critical role in a number of glaucoma phenotypes, including mitochondrial dysfunction, astrocytic activity, and synaptic plasticity [58]. RGC dendrite retraction is an early retinal response to elevated IOP that leads to functional deficits and neuronal death [10]. Dendrite retraction and synapse elimination may be mediated by innate immune responses [59] but appear to be partly reversible. Dendritic plasticity and circuit function are modulated by insulin receptor activity [60]. Pioglitazone exerts its insulin-sensitizing activity by increasing autophosphorylation of the receptor upon binding to the ligand. In mouse models of optic nerve injury, insulin administration—systemic or as eye drops—has the ability to promote regeneration of the dendrites and synapses of retinal neurons [61,62]. However, insulin signaling also supports RGC survival by decreasing neuroinflammation and altering the activity of retinal glial elements [58]. It is intriguing to speculate that pioglitazone-mediated activation of the insulin receptor intensifies the effect of the available insulin [63], leading to a similar protective effect as exogenous application.

## 5. Conclusions

In conclusion, we present data to demonstrate that oral administration of pioglitazone prevents RGC loss and reverses vision loss in two independent pre-clinical models of glaucoma. The pharmacologic activities of this well-established drug target several glaucoma pathomechanisms and it may be the convergence of these activities that result in the observed protection of RGCs.

## Data Availability

Data will be made available upon request. Please contact the corresponding author.
